# Special Issue “Probiotics, Prebiotics and Functional Foods: Health Benefits and Biosafety”

**DOI:** 10.3390/microorganisms11051218

**Published:** 2023-05-06

**Authors:** Mutamed Ayyash, Shao-Quan Liu

**Affiliations:** 1 Department of Food Science, College of Agriculture and Veterinary Medicine, United Arab Emirates University (UAEU), Al Ain P.O. Box 15551, United Arab Emirates; 2 Department of Food Science and Technology, National University of Singapore, Science Drive 2, Singapore 117543, Singapore; fstlsq@nus.edu.sg

Probiotics, prebiotics, and functional foods are buzzwords in the food industry for good reasons. These food components/ingredients offer a range of health benefits and positively impact the gut microbiome. Probiotics, live microorganisms that confer health benefits when administered in adequate amounts, have gained significant attention due to their potential in preventing and treating various diseases. On the other hand, prebiotics are non-digestible food ingredients that selectively stimulate the growth and activity of beneficial microorganisms in the gut. Functional foods, including probiotics and prebiotics, offer additional health benefits beyond their basic nutritional value.

However, as with any food product, the safety of probiotics, prebiotics, and functional foods is paramount. This Special Issue explores the latest research on these food components’ health benefits and safety. The articles in this Special Issue cover topics related to probiotics, prebiotics, and functional foods, ranging from identifying and characterizing novel probiotic strains to producing and distinguishing functional foods.

Abid et al. [1] identified and evaluated locally isolated strains of *Limosilactobacillus fermentum* from buffalo milk as potential probiotics for dairy products. They selected four strains based on their morphological and biochemical characteristics and probiotic potential, including antimicrobial activity, enzymatic potential, auto-aggregation capability, and acid and bile tolerance. The NMCC-14 strain showed the highest probiotic potential and was safe in a mouse model. Fermented milk prepared with this strain had a significantly higher protein content, water-holding capacity, and dynamic viscosity compared to non-fermented milk. The results suggest that *L. fermentum* NMCC-14 is a safe and beneficial supplement for use in the development of dairy products.

Alameri et al. [2] investigated the probiotic potential of selected lactic acid bacteria (LAB) isolated from vegetable products. The bacteria were tested for their tolerance to acid and bile, cholesterol-removing ability, resistance against lysozyme and antibiotics, production of exopolysaccharides, antimicrobial and hemolytic activities, and cell surface characteristics. The isolates showed good survival rates in the gastrointestinal tract, bile tolerance, and an ability to reduce cholesterol. They also had excellent auto-aggregation, hydrophobicity, and attachment capabilities. The identified bacteria were *Enterococcus faecium*, *E. durans*, *E. lactis*, and *Pediococcus acidilactici*.

Albedwawi et al. [3] investigated the potential of newly isolated LAB for acrylamide removal and optimized the conditions for acrylamide removal using a Box–Behnken design. *Streptococcus lutetiensis* and *Lactiplantibacillus plantarum* demonstrated the highest capability of acrylamide removal. Both strains could remove more than 30% of acrylamide at the gastric stage and around 40% at the intestinal stage under in vitro digestion conditions. The results of scanning electron microscopy coupled with energy-dispersive X-ray spectroscopy, zeta potential, transmission electron microscopy, and Fourier transform infrared spectroscopy indicated that increasing cell wall thickness improved acrylamide adsorption capacity, and functional groups C=O, C-O, and N-H were associated with acrylamide adsorption.

Aleman et al. [4] examined the effect of various functional ingredients on the probiotic characteristics, tolerance to gastric juices and lysozyme, protease activity, and viability of *Streptococcus thermophilus* and *Lactobacillus bulgaricus*. Marshmallow root, licorice root, and slippery elm bark improved the bile and acid tolerance of *S. thermophilus*. None of the tested functional ingredients affected the growth of both strains. Marshmallow root, *N*-acetyl-D-glucosamine, and maitake mushroom increased the protease activity of *S. thermophilus*. Compared to the control, marshmallow root and quercetin samples had higher mean log counts and log counts for *S. thermophilus* on the simulated gastric juice and lysozyme resistance in in vitro tests, respectively, while licorice root, quercetin, marshmallow root, and slippery elm bark samples had higher log counts for *L. bulgaricus*.

Hotchkiss et al. [5] found that cranberry-derived products contain bioactive polysaccharides and oligosaccharides, including pectic rhamnogalacturonan I, which have potential health benefits. The study characterized the structures of xyloglucan and pectic oligosaccharides from pectinase-treated cranberry pomace and demonstrated that 10 *Lactobacillus* strains can metabolize cranberry oligosaccharides as a carbon source and produce short-chain fatty acids, which are beneficial for health. These findings suggest that these strains could be potential probiotics in synbiotic products that contain cranberry oligosaccharides.

Keddar et al. [6] demonstrated that probiotic bacteria from human milk, specifically the SL42 strain, have the potential to reduce the allergic response to cow’s milk casein in rats. The SL42 strain decreased histamine levels, milk casein-specific IgE levels, eosinophil numbers, S100A8/9 levels, and cytokine concentrations, while also increasing LAB and *Clostridium* species in the gut microbiota. Histological analysis of the jejunum confirmed the protective effect of the probiotic bacteria. These findings suggest that probiotics from human milk may play a role in ameliorating cow’s milk casein allergy.

Kim et al. [7] investigated the effects of heat-killed *Bifidobacterium* and *Lactobacillus* strains on human colorectal carcinoma RKO cells in in vitro and in vivo xenograft models. The results showed that *B. bifidum* MG731, *L. reuteri* MG5346, and *L. casei* MG4584 had strong cytotoxic and apoptotic effects on RKO cells. Oral administration of these strains significantly delayed tumor growth, and a combination of MG5346 and MG4584 or MG731, MG5346, and MG4584 synergistically inhibited tumor growth in the xenograft model. The study suggests that the combination of MG5346 and MG4584 could be effective as parabiotics or postbiotics in inhibiting colorectal cancer growth.

Lee et al. [8] investigated the potential protective effects of *Bifidobacterium animalis* subsp. *lactis* MG741 (*B. lactis* MG741) against skin photoaging induced by UVB exposure. The results showed that *B. lactis* MG741 reduced wrinkles and skin thickness by downregulating MMP-1 and MMP-3, phosphorylation of ERK, and c-FOS in fibroblasts and hairless mice. It also inhibited the expression of NF-κB, an inflammation-related factor in UVB-irradiated dorsal skin. Therefore, *B. lactis* MG741 has the potential to prevent wrinkles and skin inflammation by modulating skin photoaging markers.

Maftei et al. [9] focused on creating a symbiotic beverage product that combines soy milk with sea buckthorn syrup or powder and inulin, fermented with a strain of *Lacticaseibacillus paracasei* to provide health benefits, such as probiotics, prebiotics, and minerals. The research tested the survivability of probiotic bacteria, pH, and titratable acidity during the fermentation period and determined probiotic viability, pH, titratable acidity, and water-holding capacity during storage for 14 days at 4 °C. The results showed that novel symbiotic beverages based on sea buckthorn syrup or powder, inulin, and soy milk were successfully produced, offering microbiological safety and excellent sensory attributes.

Maillard et al. [10] proposed a new flowchart for identifying probiotic strains that have potential in the prevention or treatment of IBD and IBS. The flowchart included in vitro tests of immunomodulatory properties, assessment of the barrier-strengthening effect, and quantification of SCFAs and AhR agonists produced by the strains. The study tested this flowchart on a collection of 39 LAB and *Bifidobacterium* strains and identified two promising strains associated with an anti-inflammatory profile. These strains were then validated in mouse models of post-infectious IBS or chemically induced colitis to mimic IBD. The study’s results suggest that this screening strategy can effectively identify strains with potential beneficial effects on colonic inflammation and hypersensitivity.

Memon et al. [11] investigated the effect of *Bacillus subtilis* (*B. subtilis*) probiotic feeding on the chicken gut microbiota response to Eimeria infection, which causes coccidiosis. The results showed that probiotic supplementation improved growth performance, enhanced immunity, maintained gut homeostasis, and modulated the gut microbiota during enteric infection. The supplementation of probiotic *B. subtilis* positively influenced the abundance of some commensal genera, thereby alleviating the Eimeria-induced intestinal disruption. Overall, these findings suggest that probiotic supplementation could play a key role in preventing and managing coccidiosis in poultry.

Nikolaou et al. [12] investigated freeze-dried Lacticaseibacillus rhamnosus OLXAL-1 cells, free and immobilized on apple pieces, to fortify grape juice. The results showed that refrigerated storage resulted in cell loads > 7 log cfu/g for both free and immobilized cells for up to 10 days, achieving populations > 10^9^ cfu per share, with no noticeable spoilage. Immobilized cells resulted in a significant growth limitation of food-spoilage microorganisms compared to unfortified juice. HS-SPME GC/MS analysis revealed that both the nature of the freeze-dried cells and storage temperature significantly affected the content of minor volatiles detected, resulting in significant differences in the total volatile concentration. The fortified juice products were accepted during the preliminary sensory evaluation, and the authors perceived juices with freeze-dried immobilized cells as highly novel.
